# Advances in the Synthesis of Heteroaromatic Hybrid Chalcones

**DOI:** 10.3390/molecules28073201

**Published:** 2023-04-04

**Authors:** Ajay Mallia, Joseph Sloop

**Affiliations:** Department of Chemistry, School of Science & Technology, Georgia Gwinnett College, 1000 University Center Lane, Lawrenceville, GA 30043, USA

**Keywords:** chalcone, heteroaromatic, hybrid chalcone

## Abstract

Chalcones continue to occupy a venerated status as scaffolds for the construction of a variety of heterocyclic molecules with medicinal and industrial properties. Syntheses of hybrid chalcones featuring heteroaromatic components, especially those methods utilizing green chemistry principles, are important additions to the preparative methodologies for this valuable class of molecules. This review outlines the advances made in the last few decades toward the incorporation of heteroaromatic components in the construction of hybrid chalcones and highlights examples of environmentally responsible processes employed in their preparation.

## 1. Introduction

**Figure 1:**
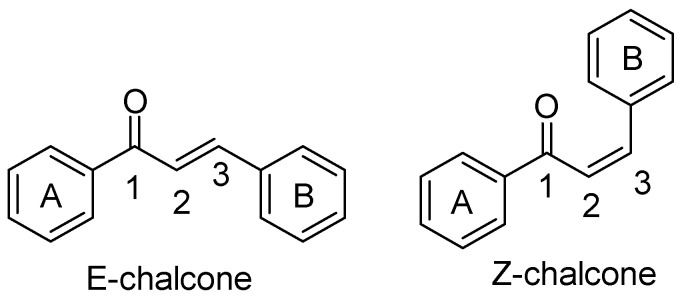
Chalcone structure and stereochemistry.

The chalcone class of enones has been a privileged scaffold in organic synthesis for more than a century. Kostanecki and Tambor are credited with the first reported preparation of E-1,3-diphenylprop-2-en-1-one and coined the term “chalcone” in 1899 [1]. Figure 1 shows the structure of E-chalcone, the most energetically favorable stereoisomer, as well as the sterically encumbered and less common Z-chalcone, both of which contain benzene rings at C_1_ and C_3_ joined by a three-carbon α,β-unsaturated ketone unit. The absolute configuration of solid chalcone stereochemistry obtained during synthesis can often be determined with X-ray crystallography [2,3].

By convention, the aromatic ring attached to C_1_ is designated as ring A while the aromatic ring attached to C_3_ is designated as ring B. For the purposes of this review, we will adhere to the conventional ring designations in describing preparations of heteroaromatic hybrid chalcones.

The utility of chalcones both as a pharmacophore and as a scaffold in the synthesis of a wide variety of heterocycles ranging from pyrazoles, isoxazoles, triazoles, barbituric acid derivatives, etc. has been investigated thoroughly over the years, with numerous research articles as well as several reviews appearing in the last decade describing the current chalcone synthetic strategies, the heterocycles derived from them, and the bioactivity and pharmaceutical uses of these compounds [4,5,6,7,8,9,10,11,12,13]. Within that context, the preparation of more highly functionalized chalcones that contain heteroaromatic components has been an area of intense research over the last decade [10,14,15,16,17,18,19,20,21,22,23,24,25,26,27,28,29,30].

**Figure 2:**
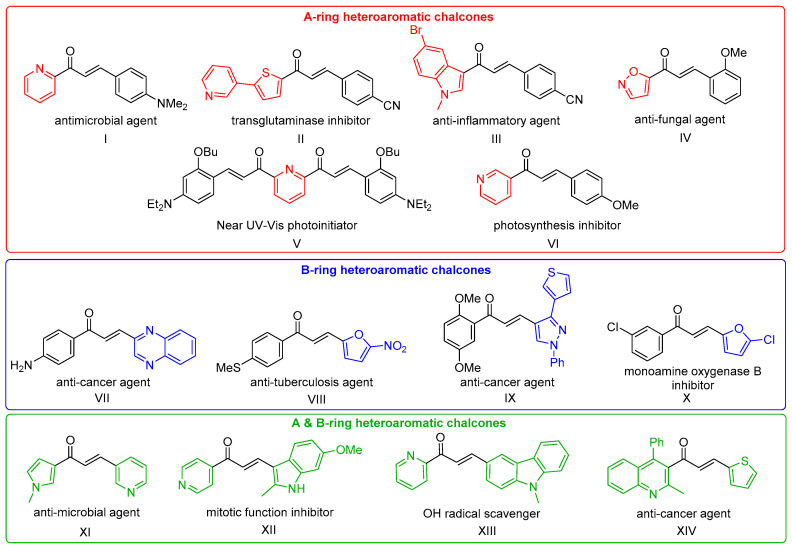
Medicinally and industrially important heteroaromatic hybrid chalcones.

Research has established that heteroaromatic hybrid chalcones themselves possess broad medicinal value as anticancer [16,19,23], antimicrobial [11,20,23,28], antifungal [16], anti-tuberculosis [25] and anti-inflammatory agents [22] as well as having other important pharmacological functions [9,10], agrochemical utility as photosynthesis inhibitors [18] and industrial use as photoinitiators in 3D printing [17]. Figure 2 shows a representative selection of heteroaromatic hybrid chalcone pharmacophores and industrially important compounds.

**Figure 3:**
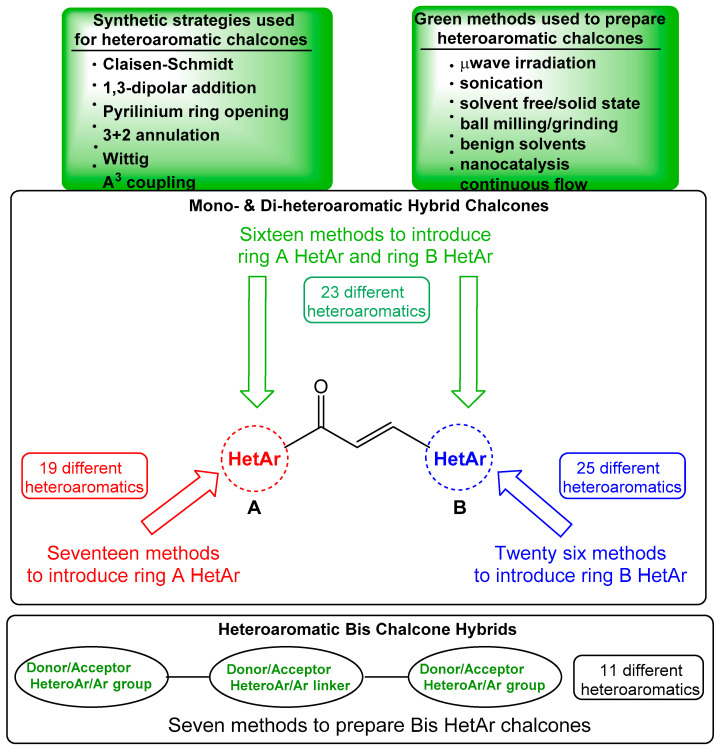
Heteroaromatic hybrid chalcone construction.

Synthetic methodologies to prepare hybrid chalcones have developed rapidly over the last two decades. To the best of our knowledge, no reviews have been found that focus on heteroaromatic chalcone synthesis and the green synthesis methods employed to prepare them. This review will focus on the construction of heteroaromatic hybrid chalcones with the Claisen–Schmidt condensation, 1,3-dipolar additions, ring-opening reactions, 3+2 annulations and Wittig reactions. The review will discuss four different heteroaromatic hybrid chalcone types: A-ring and B-ring-substituted mono-heteroaromatic hybrid chalcones, hybrid chalcones possessing heteroaromatic moieties on both the A and B rings, and the synthesis strategies used to prepare heteroaromatic bis chalcone hybrids. Herein, we also detail the green methods that have been employed to prepare these hybrid chalcones including microwave irradiation, sonication, ball milling, continuous flow reactions, the use of benign solvents, solvent-free/solid-state processes and nanocatalysis. See Figure 3.

## 2. A-Ring Heteroaromatic Hybrid Chalcone Synthesis

This section catalogues several representative conventional and green processes by which hybrid chalcones bearing a heteroaromatic species at ring A may be prepared. Heteroaromatic components of the chalcone products include a variety of single-ring (furan, pyrrole, thiazole, thiophene, pyridine, pyrimidine) and fused-ring (indole, benzimidazole, benzothiazole, benzofuran, pyrazolopyridine, quinoline) systems.

### 2.1. Claisen–Schmidt Condensations

The Claisen–Schmidt (C-S) condensation has been widely used to prepare chalcones for many years. This reaction, which can be catalyzed by acids or bases, offers mild conditions that tolerate a wide scope of functionality in both the ketone donors and aldehyde acceptors.

#### 2.1.1. Base-Catalyzed C-S Condensations

**Scheme 1:**
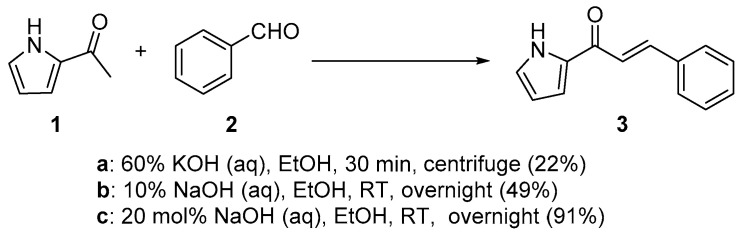
Synthesis of pyrrolyl chalcone.

**Scheme 2:**
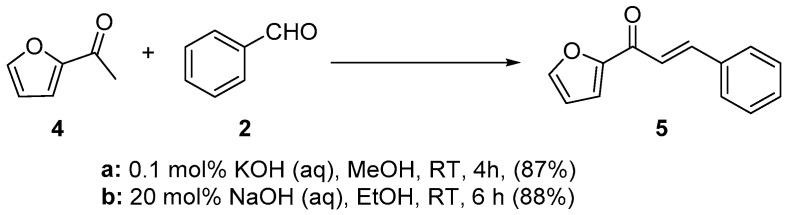
Synthesis of furyl chalcone.

**Scheme 3:**
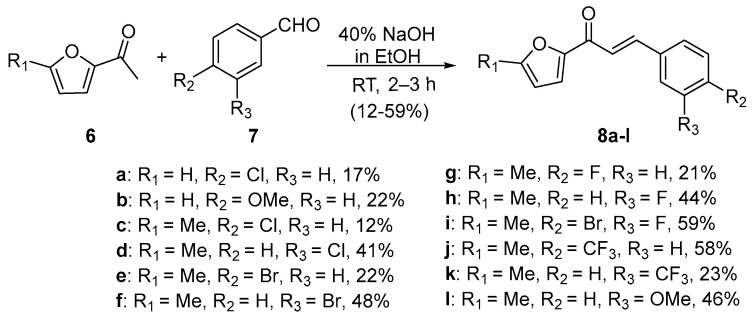
Synthesis of furyl chalcone derivatives.

**Scheme 4:**
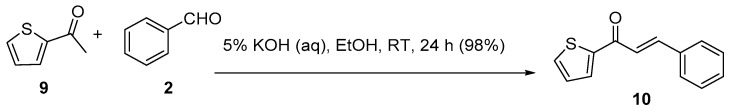
Synthesis of thienyl chalcone.

**Scheme 5:**
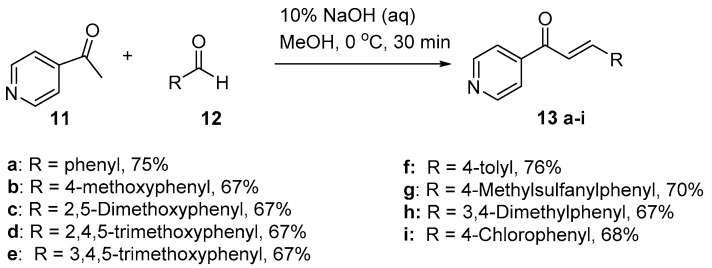
Synthesis of pyridyl chalcone.

**Scheme 6:**
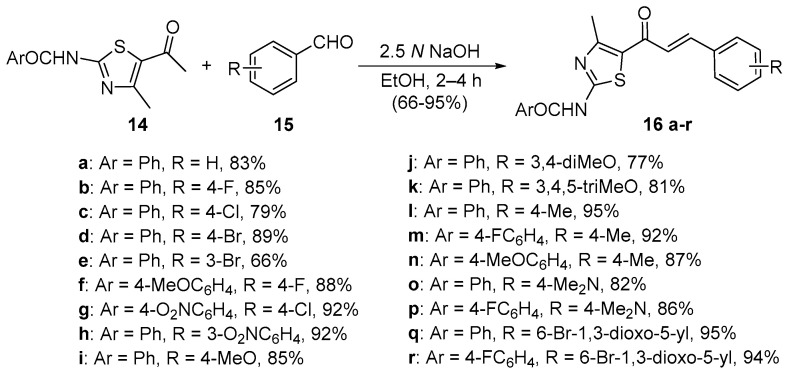
Synthesis of thiazolyl chalcones.

**Scheme 7:**
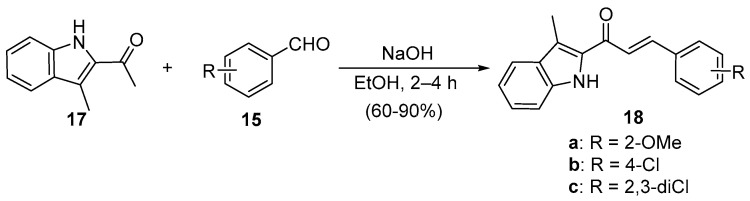
Synthesis of indolyl chalcones.

**Scheme 8:**
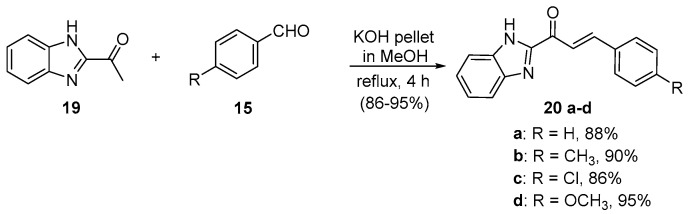
Synthesis of benzimidazolyl chalcones.

**Scheme 9:**
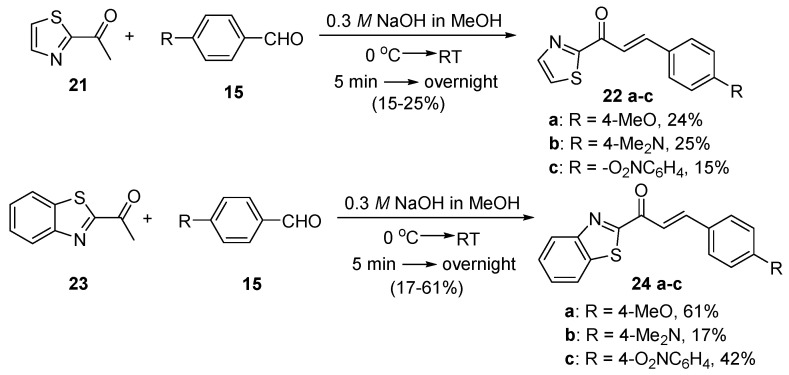
Synthesis of thiazolyl and benzothiazolyl chalcones.

**Scheme 10:**
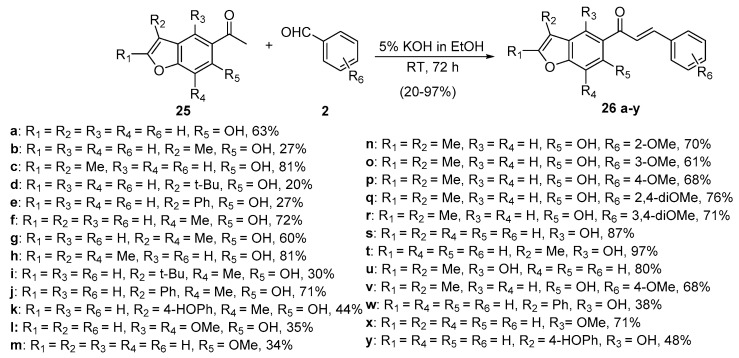
Synthesis of benzofuryl chalcones.

**Scheme 11:**
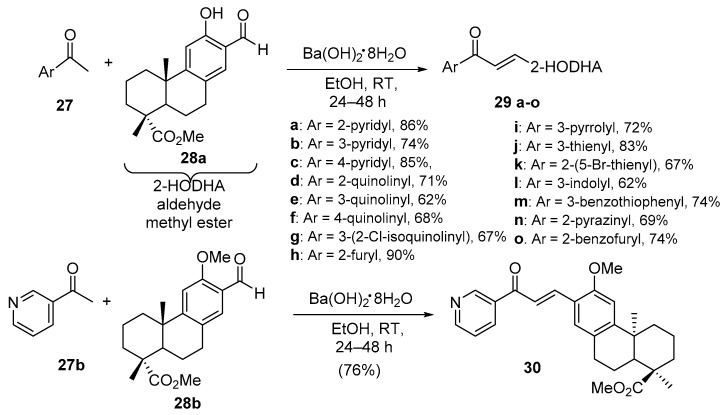
Synthesis of heteroaromatic dehydroabietic acid-chalcone hybrids.

**Scheme 12:**
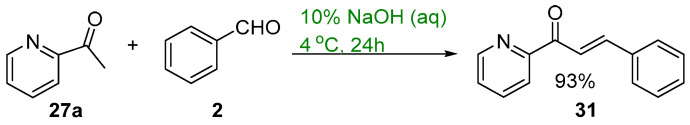
Green synthesis of pyridyl chalcone.

The hydroxide bases KOH, NaOH and to a lesser extent Ba(OH)_2_ are the bases used to promote the condensations depicted below in Scheme 1, Scheme 2, Scheme 3, Scheme 4, Scheme 5, Scheme 6, Scheme 7, Scheme 8, Scheme 9, Scheme 10, Scheme 11 and Scheme 12. These bases may be introduced to the reaction medium as dilute or concentrated aqueous solutions or as solids. Ethanol or methanol are the solvents of choice in most reactions depicted herein. The reaction temperatures vary from 0 °C to those obtained by refluxing the alcoholic solvents. The reaction times range from less than a minute in the case of selected microwave-mediated reactions and can extend to 72 h for the conventional condensations.

In our first entry, three room-temperature C-S preparations of pyrrolyl chalcone **3** are presented that have differing reaction times and different base concentrations. Sweeting et al. (Scheme 1a) used strongly basic conditions (60% aqueous KOH) and centrifugation mixing to prepare the pyrrolyl chalcone **3** in a modest yield. The low yield is likely attributed to the short reaction time. Ref. [31] Robinson et al. reported that increasing the reaction time ([32], Scheme 1b) using NaOH (aq) in ethanol increased the yield of the pyrrolyl chalcone. Using 20 mol % NaOH (aq) in ethanol, Song et al. obtained a 91% yield in the preparation of the chalcone (Scheme 1c). Ref. [33] Lokeshwari’s team (Scheme 2a) and Liu’s group prepared furyl chalone **5** in an 87% yield using 0.1 mol % KOH (aq) in 4 h, while Liu’s group (Scheme 2b) obtained equally high yields with 20 mol% NaOH (aq) in 6 h [34,35]. Robinson et al. (Scheme 3) condensed 2-acetylfuran and 2-acetyl-5-methylfuran with assorted benzaldehydes at room temperature en route to the twelve furyl chalcones **8** in modest to medium yields [36]. 

Parveen et al. reported a nearly quantitative conversion for the room-temperature C-S condensation of 2-acetylthiophene and benzaldehyde using aqueous KOH (Scheme 4) in ethanol to the thienyl chalcone **10** [37].

Sunduru et al. reported the preparation of pyridyl chalcone derivatives **13** by condensing 4-acetylpyridine with the respective aromatic aldehyde (Scheme 5) [38]. In this reaction, one equivalent of 4-acetylpyridine was added dropwise to a cooled methanolic solution containing 10% aqueous NaOH. Then, one equivalent of aldehyde was added slowly at 0 °C. After workup and recrystallization, the pyridyl chalcones were obtained in yields ranging from 67 to 76% (Scheme 5).

Sinha and coworkers (Scheme 6) used similar conditions to synthesize eighteen 1,3-thiazolylchalcones **16** in very good overall yields [39]. 

Zhao et al. (Scheme 7) used reflux conditions to achieve yields in excess of 60% for the small series of fused-ring indolyl chalcones **18** [40]. In two separate publications, Hsieh and coworkers used base-catalyzed C-S condensations to prepare indolyl (Scheme 8, [41]), thiazolyl and benzothiazolyl hybrid chalcones (Scheme 9, [42]). 

Saito’s team used 5% KOH in ethanol at room temperature to prepare a series of functionalized benzofuran hybrid chalcones in yields as high as 97% (Scheme 10) [43]. 

Grigoropoulou’s team found barium hydroxide octahydrate effective in promoting the condensation of both single- and fused-ring heteroaromatic ketones with dehydroabietic acid methyl ester en route to sixteen hybrid chalcones in good overall yields (Scheme 11) [44].

Base-catalyzed C-S condensations have also been demonstrated using green principles. These processes include the use of benign solvents including water and microwave irradiation. Mubofu and Engberts reported a C-S condensation reaction of 2-acetylpyridine and benzaldehyde using 10% NaOH (Scheme 12) [45]. The reagents were finely dispersed in water at 4 °C and after workup the pyridyl chalcone **31** was obtained in a good yield (Scheme 12). 

**Scheme 13:**
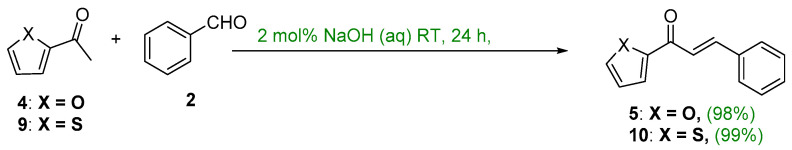
Green synthesis of furyl and thienyl chalcones.

Jianga et al. showed that the condensation of 2-acetylfuran or 2-acetylthiophene and benzaldehyde using 2 mol% NaOH (aq) gave (E)-1-(Furan-2-yl)-3-phenylprop-2-en-1-one or (E)-1-(thiophen-2-yl)-3-phenylprop-2-en-1-one at room temperature in nearly quantitative yields (Scheme 13) [46].

**Scheme 14:**
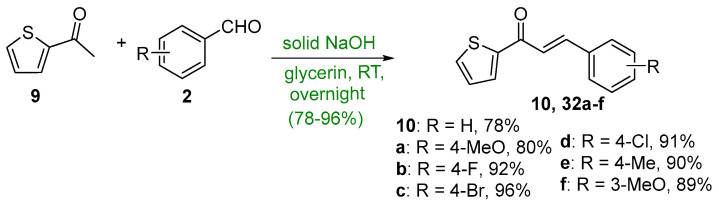
Green synthesis of 2-thienyl chalcones.

Ritter et al. (Scheme 14) used 2-acetylthiophene **9** and assorted benzaldehydes in glycerin solvent to prepare seven 2-thienochalcones **10** and **32a–f** in very good yields [47]. 

**Scheme 15:**
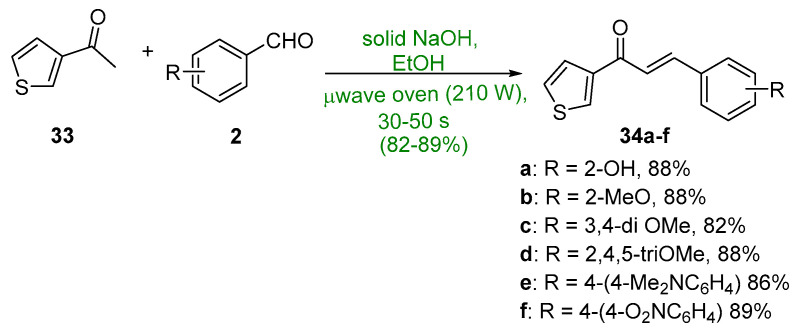
Microwave synthesis of 3-thienyl chalcones.

Khan and Asiri (Scheme 15) showed that 3-acetylthiophene **33** underwent a microwave-mediated C-S condensation with several benzaldehydes in less than a minute to give thienyl chalcones **34a–f** in yields exceeding 82% [48]. 

**Scheme 16:**
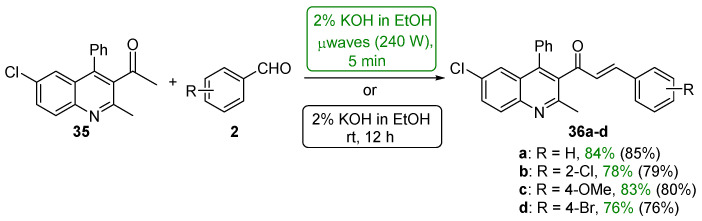
Synthesis of quinolinyl chalcones.

Sarveswari and Vijayakumar (Scheme 16) conducted a comparative study of conventional and microwave processes in which four examples of highly substituted quinolinyl hybrid chalcones **36a–d** were prepared [49]. Both processes gave the desired chalcones in yields greater than 75%. Particularly noteworthy is the fact that the microwave reaction time is 1/144 of the conventional reaction time.

**Scheme 17:**
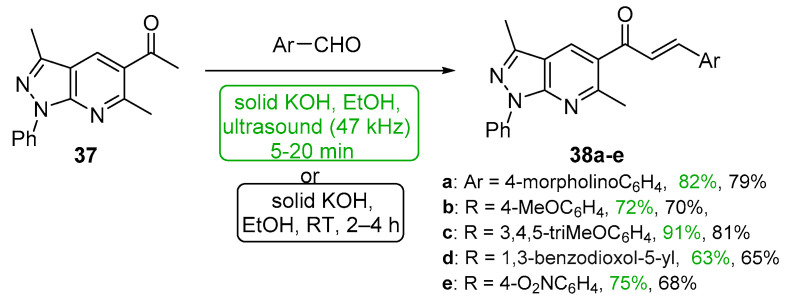
Sonochemical synthesis of pyrazolopyridyl chalcones.

Polo et al. demonstrated that sonochemical mediation was very effective in preparing a series of pyrazolopyridyl hybrid chalcones **38a–e** (Scheme 17) in high yields that compare favorably with conventional base-catalyzed C-S condensations [50].

#### 2.1.2. Acid-Catalyzed C-S Condensations 

**Scheme 18:**
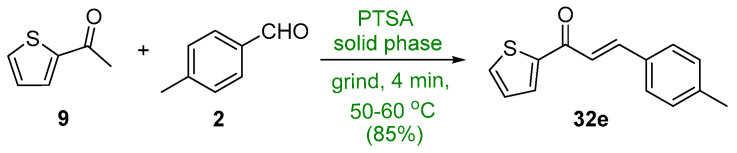
PTSA-catalyzed synthesis of thienyl chalcone.

In the recent literature, Adnan et al. showed that *p*-toluenesulfonic acid (PTSA) effectively catalyzed the condensation of 2-acetylthiophene (**9**) and *p*-tolualdehyde (**2**) in a green solventless process in which the reactants were ground in a warm mortar and pestle for 4 min to give the thienyl chalcone **32e** in a very good yield [13]. See Scheme 18.

**Scheme 19:**
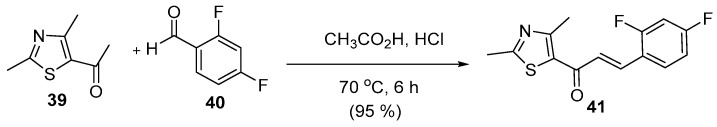
Acid-catalyzed synthesis of thiazolyl chalcone.

Shaik et al. reported an acid-catalyzed condensation reaction of 2,4-dimethyl-5-acetylthiazole with 2,4-difluorobenzaldehyde to prepare (E)-1-(2′,4′-dimethyl)-(5-acetylthiazole)-(2,4″-difluorophenyl)-prop-2-en-1-one (Scheme 19) [23].

### 2.2. Non C-S Condensations 

**Scheme 20:**
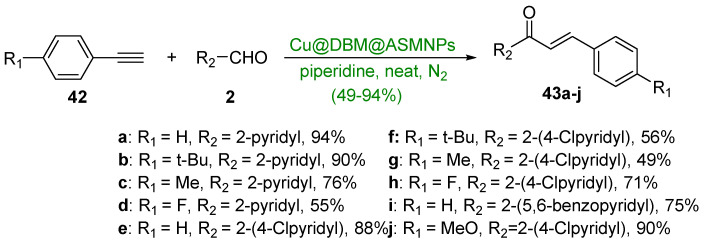
Cu-based nanocatalyzed A^3^ synthesis of pyridyl- and benzopyridyl chalcones.

Our final installment of A-ring hybrid chalcone synthesis is an interesting green coupling reaction between a series of arylacetylene derivatives (**42a–j**) and various pyridine and benzopyridine carboxaldehydes (Scheme 20). Yadav’s group showed that a copper-based silica-coated magnetic nanocatalyst (Cu@DBM@ASMNPs) used in conjunction with a piperidine base was very effective in preparing ten hybrid chalcones in yields ranging from 49 to 94% [51]. A noteworthy feature of this reaction was the ability to recover the catalyst via a magnet. The catalyst was reported to be efficient for up to seven reaction cycles.

## 3. B-Ring Heteroaromatic Hybrid Chalcone Synthesis

This section catalogues selected conventional and green processes by which hybrid chalcones containing a heteroaromatic component at ring B may be prepared. In addition, examples of tandem ring-opening dipolar additions to obtain ring B heteroaromatic substituted chalcones are presented. The heteroaromatic components of the chalcone products highlighted in this section include a variety of single-ring (furan, pyrrole, pyrazole, thiazole, thiophene, pyridine) and fused-ring (indole, benzimidazole, benzothiazole, benzofuran, quinoline, imidazo [1,2-a]pyrimidine or imidazo [1,2-a]pyridine, quinoxaline, carbazole) systems.

### 3.1. Claisen–Schmidt Condensations 

As in the preceding section, Claisen–Schmidt (C-S) condensation has been widely used to prepare B-ring heteroaromatic chalcones. This reaction, which can be catalyzed by bases or acids, offers mild conditions that tolerate a wide scope of functionality in both the ketone donors and aldehyde acceptors.

#### Base-Catalyzed C-S Condensations

**Scheme 21:**
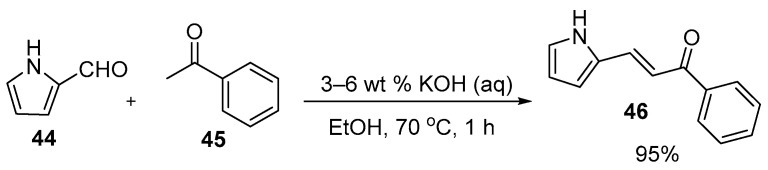
Pyrrolyl chalcone synthesis.

**Scheme 22:**
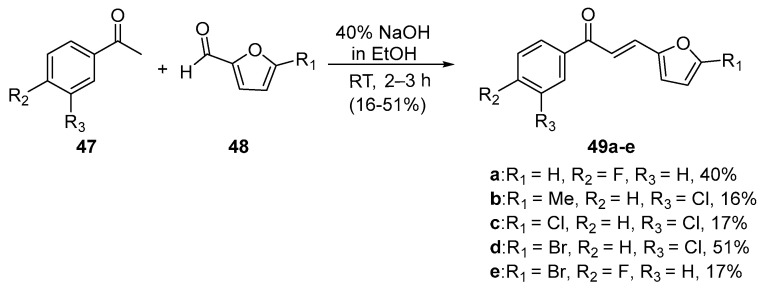
Furyl chalcone synthesis.

In the preparations shown below, NaOH and KOH are the bases of choice. Shown in Scheme 21, Li et al. used dilute aqueous KOH to prepare pyrrolyl chalcone (**46**) in a very good yield. Using mild conditions, Robinson et al. (Scheme 22) condensed acetophenones **47** and furfural derivatives **48** to prepare five furyl chalcones (**49a–e**) that show promise as monoamine oxidase inhibitors in low to medium yields [36].

**Scheme 23:**
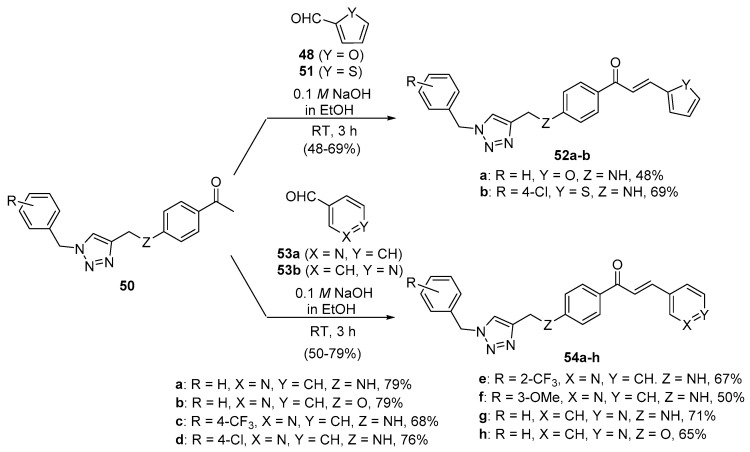
Furyl, thienyl and pyridyl chalcone synthesis.

In Scheme 23, Fu and coworkers reacted 1,2,3-triazole-substituted acetophenones **50** with furfural **48** and thiophene-2-carbaldehyde **51** in ethanolic KOH for 3 h to prepare hybrid chalcones **52a** and **52b** in satisfactory yields. Condensation of **50** and pyridine carbaldehydes **53a–b** under the same conditions provided eight additional pyridyl hybrid chalcone examples **54a–h** in yields ranging from 50 to 79% [52].

**Scheme 24:**
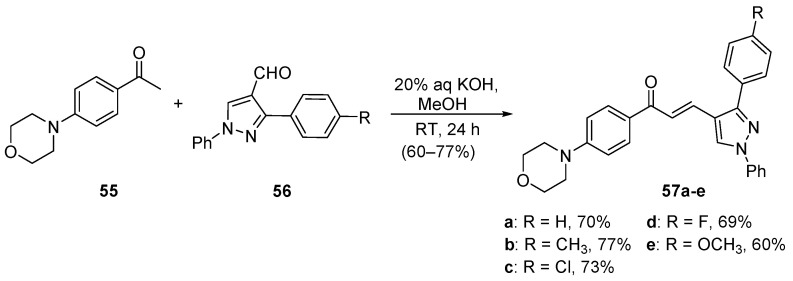
Pyrazolyl chalcone synthesis.

Gadhave and Uphade demonstrated the satisfactory condensation of 4-morpholinoacetophenone **55** with 4-pyrazolocarbaldehydes **56** conducted at room temperature, which provided five examples of 4-pyrazolylchalcones **57** [53]. See Scheme 24.

**Scheme 25:**
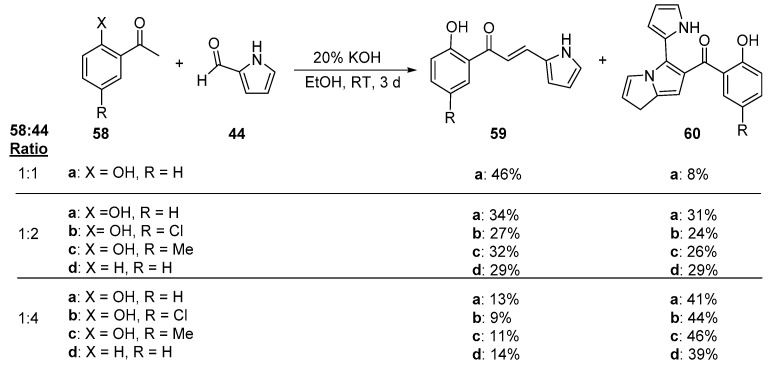
Pyrrolyl chalcone synthesis.

An interesting study conducted by Mallik and associates involves the preparation of pyrrole-substituted hybrid chalcones from the C-S condensation of several acetophenones **58** and 2-formylpyrrole **44** under different molar ratios of **58**:**44** [54]. As Scheme 25 shows, the desired product **59** predominated when the reactant molar ratios were 1:1, but when the ratio was lowered to 1:2, a nearly equal proportion of the product mixture was found to be the heteroaromatic ketone **60**. Upon increasing the molar proportion of **58** to four times that of **44**, ketone **60** was the major product. The authors propose an interesting mechanism by which **60** is formed—a twin aldol addition—intramolecular cyclization-dehydration.

**Scheme 26:**
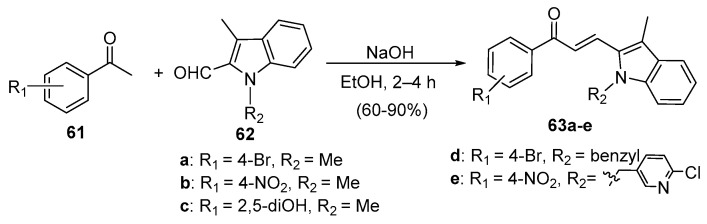
Indolyl chalcone synthesis.

Fused-ring heteroaromatic aldehydes have also been successfully condensed with various acetophenones to prepare B-ring hybrid chalcones under typical C-S reaction conditions. Zhao et al. prepared indole hybrid chalcones **63a–e** (Scheme 26) from assorted acetophenones and N-methylindolycarbaldehydes **62** in yields ranging from 60 to 90% [40].

**Scheme 27:**
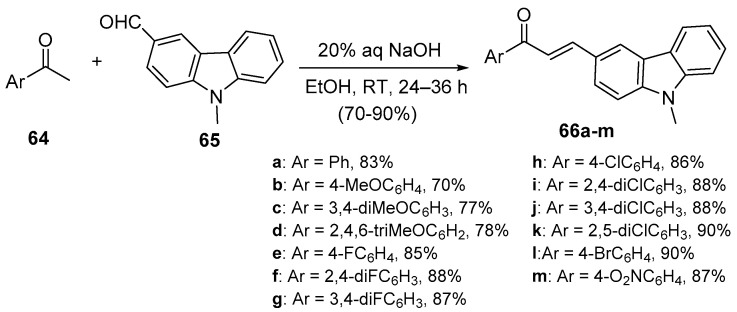
Carbazolyl hybrid chalcone synthesis.

**Scheme 28:**
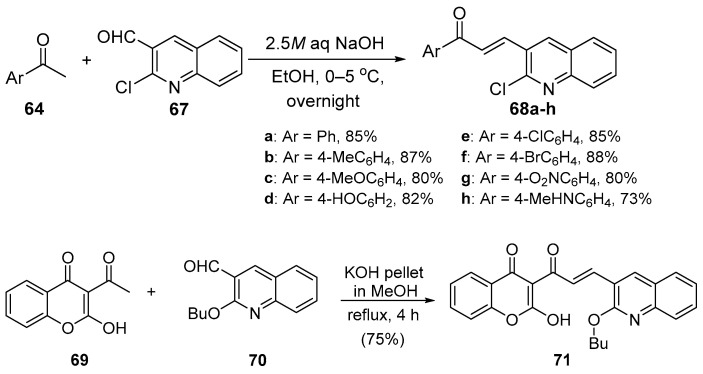
Quinolinyl hybrid chalcone synthesis.

**Scheme 29:**
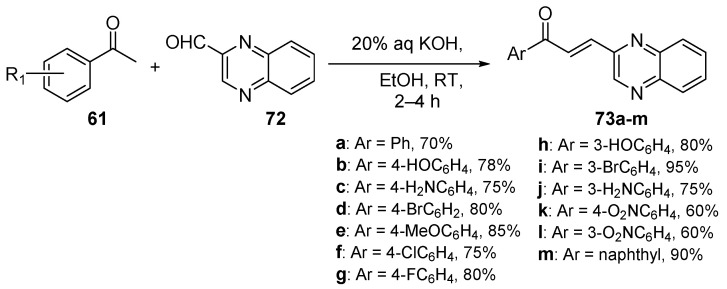
Quinoxalinyl hybrid chalcone synthesis.

Bandgar and coworkers (Scheme 27) synthesized a diverse library of carbazole hybrid chalcones **66** [30], while Bindu’s team condensed acetophenone derivatives with quinoline carboxaldehdes **68** under mild C-S conditions (Scheme 28) to prepare eight examples of B-ring-substituted quinolinoid hybrid chalcones **68a–h** [55]. Abonia et al. prepared the chromen-4-one—quinoline hybrid chalcone **71** under similar conditions [56]. See Scheme 29.

Desai and coworkers used mild C-S reaction conditions to prepare a series of thirteen quinoxalinyl hybrid chalcones **73a–m** in yields ranging from 60 to 95%, as shown in Scheme 29 [24].

**Figure 4:**
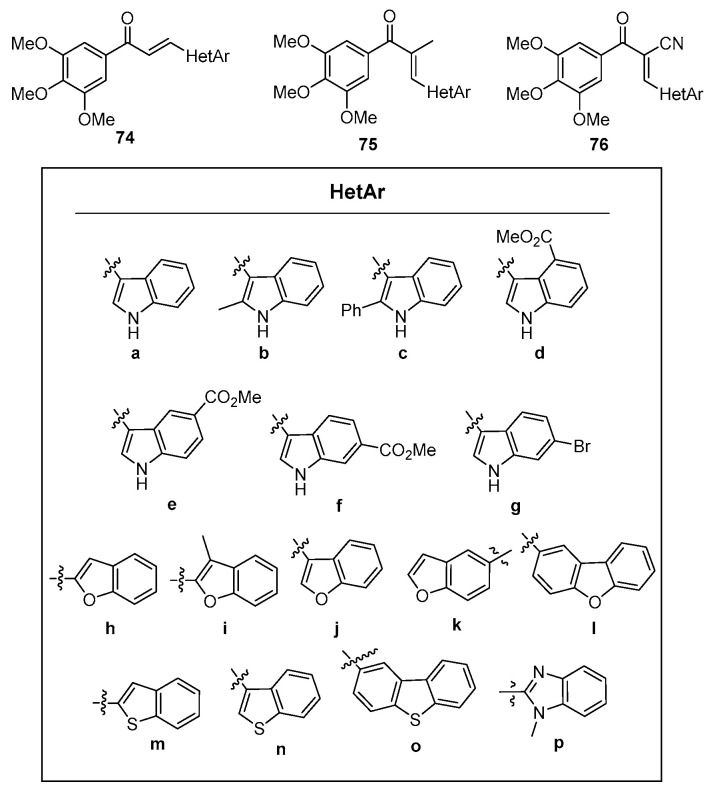
Hybrid chalcone heteroaromatic components prepared by Sun et al.

**Scheme 30:**
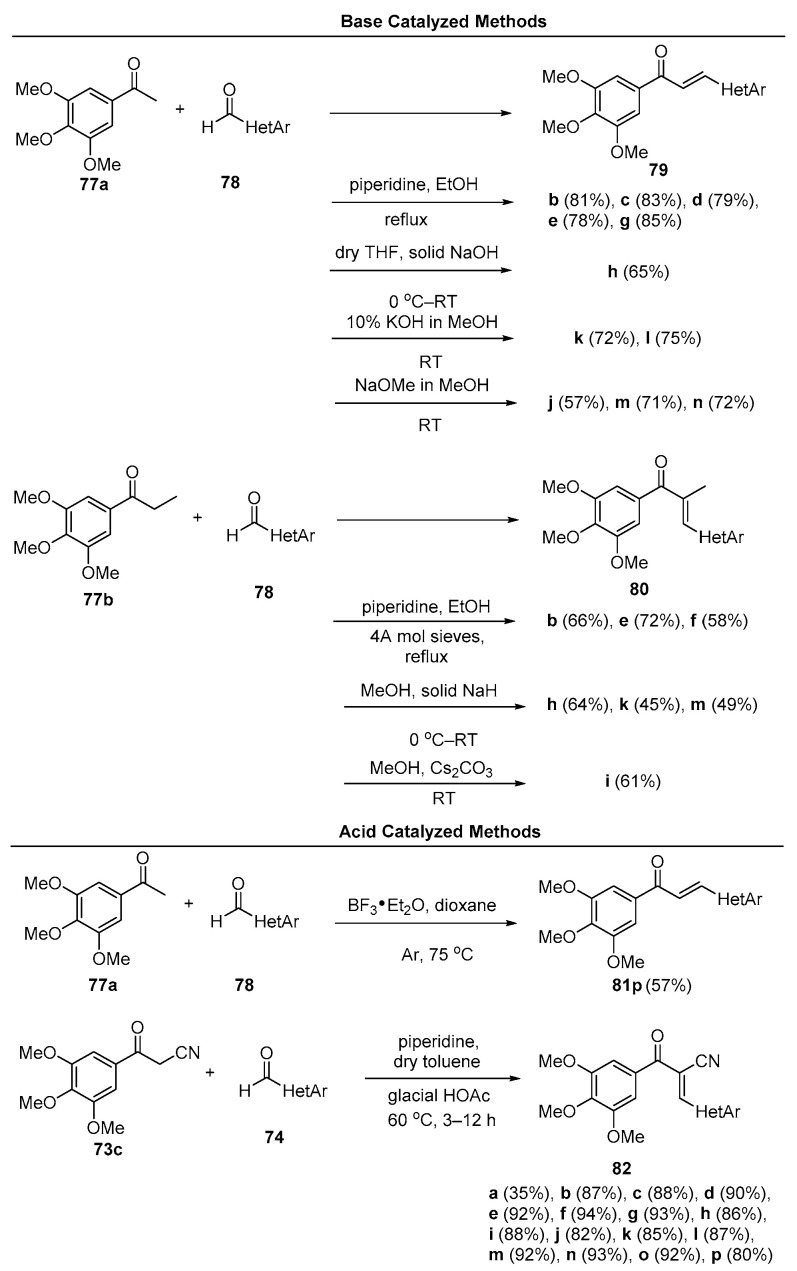
N, O, S Fused-ring heteroaromatic hybrid chalcone synthesis.

In a study of microtubule polymerization inhibition, Sun et al. synthesized a library of fused-ring heteroaromatic chalcones featuring indoles, benzofurans, dibenzofurans, benzothiophenes, dibenzothiophenes, and benzimidazoles [57]. See Figure 4. Of particular note were the numerous methods used in the preparation of these hybrid chalcones, which included both base-promoted processes (piperidine, NaOH, KOH, NaOMe, Cs_2_CO_3_ and NaH) in methanolic and ethanolic solvents, Lewis acid catalysis (BF_3_•etherate) in dioxane solvent and Brønsted (glacial acetic acid) acid catalysis in toluene. Scheme 30 depicts the scope of this work.

**Scheme 31:**
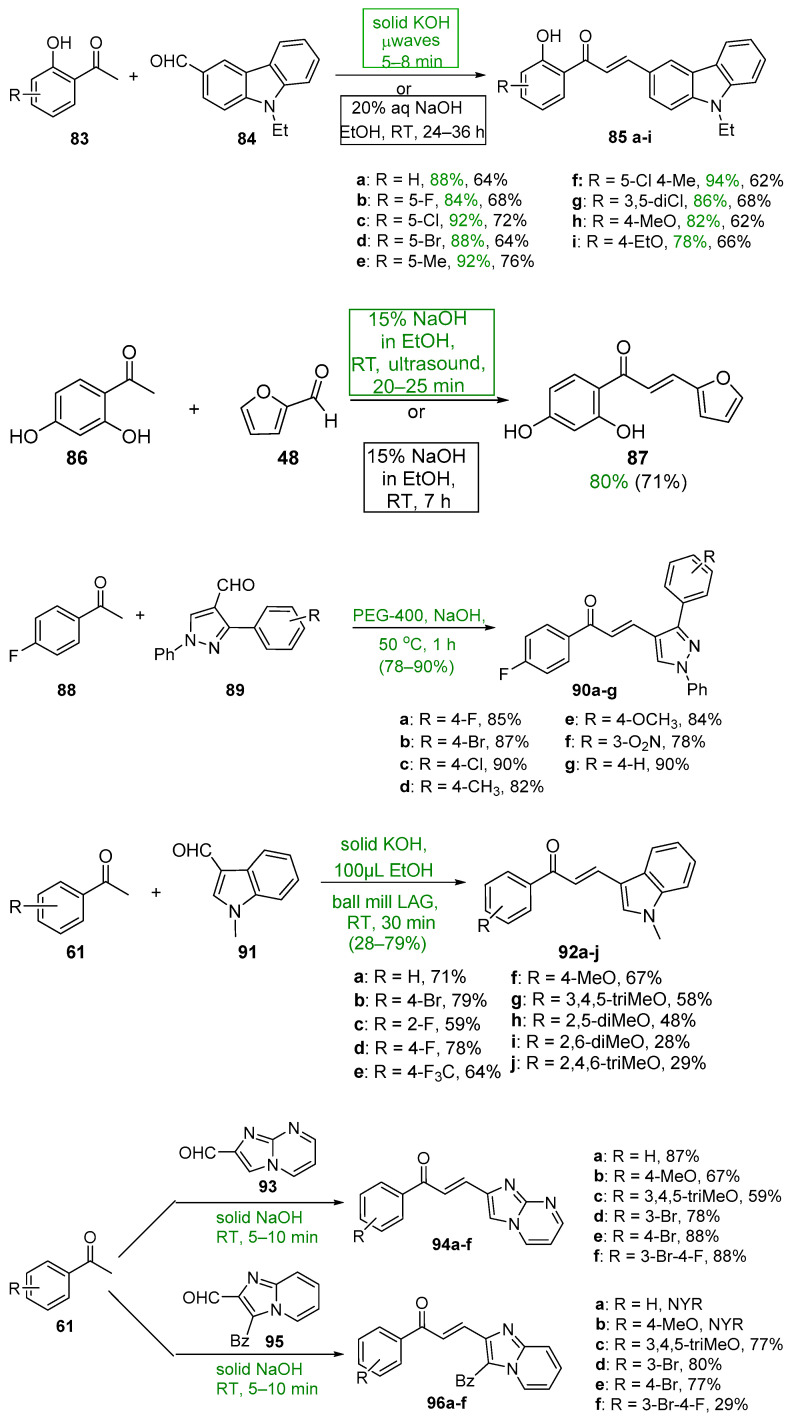

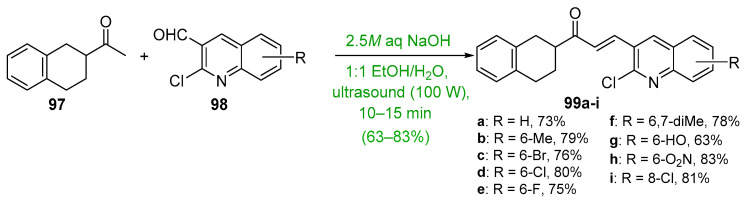
Green syntheses of B-ring heteroaromatic hybrid chalcones.

Base-catalyzed C-S condensations that employ green chemistry principles to produce B-ring-substituted hybrid chalcones have also been successfully conducted. See Scheme 31. These processes include the use of benign solvents, solvent-free reactions, microwave irradiation, ultrasound and ball milling. For example, Ashok’s group compared a typical base-catalyzed C-S condensation of **83** and **84** with a solvent-free, microwave-mediated process to prepare a series of carbazolyl hybrid chalcones **85** [58]. The yields for the short-duration microwave-mediated reactions exceeded those of the lengthy conventional C-S reactions in every case. Bhatt et al. prepared the furyl chalcone **87** using both conventional C-S and ultrasound processes to condense furfural **48** and 2,4-dihydroxyacetophenone **86** [59]. The effectiveness of sonication is evident—a 10% increase in yield in 1/20 the reaction time. Jadhava’s team used PEG-400 as a benign solvent to mediate the condensation of 4-fluoroacetophenone 84 and a series of pyrazole carbaldehydes **85** en route to eight fluorinated pyrazolyl hybrid chalcones 86 [60]. Kudlickova and coworkers employed a mechanochemical ball-milling process to prepare a series of indoylchalcones **92** in yields ranging from 28 to 79% in only 30 min [61]. Nimmala’s group used a solventless process to condense various acetophenones and imidazo [1,2-a]pyrimidine **93** or imidazo [1,2-a]pyridine **95** en route to hybrid chalcones **94a–f** and **96a–f**, respectively, in very good yields [62]. Joshi and Saglani employed ultrasound to assist in the condensation of the fused-ring ketone **97** and a series of quinoline carbaldehydes **98** to prepare the quinolinyl hybrid chalcones **99** [63].

### 3.2. Non C-S Condensations 

**Scheme 32:**
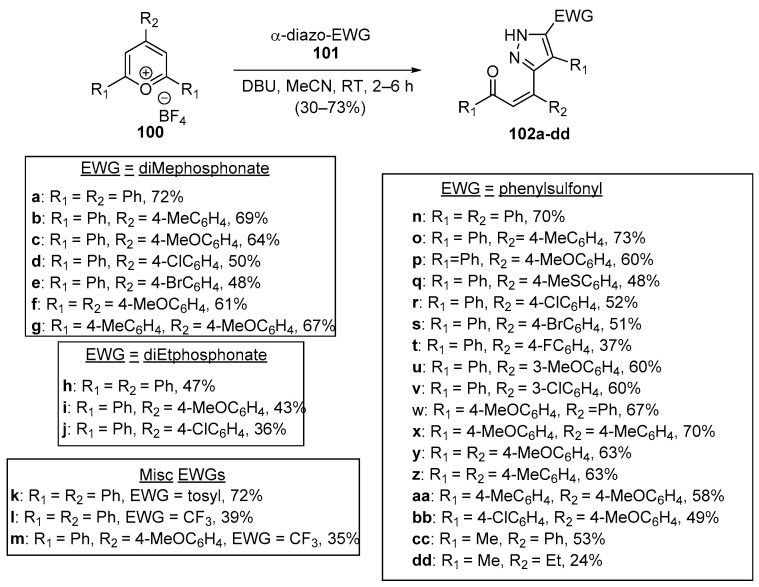
Synthesis of pyrazole hybrid Z-chalcones via a pyrilium.ring-opening dipolar addition.

The final entries describing ring-B-substituted heteroaromatic hybrid chalcones feature unique tandem reactions involving pyrylium tetrafluoroborate derivatives. Devi and colleagues conducted a very interesting examination of a single-pot, base-mediated, tandem-ring-opening, 1,3-dipolar addition reaction between several electron withdrawing group (EWG)-substituted diazo compounds **101** with tri-substituted pyrylium salts **100**, producing an extensive array of pyrazole hybrid chalcones **102** in moderate to high yields, as shown in Scheme 32 [64].

**Scheme 33:**
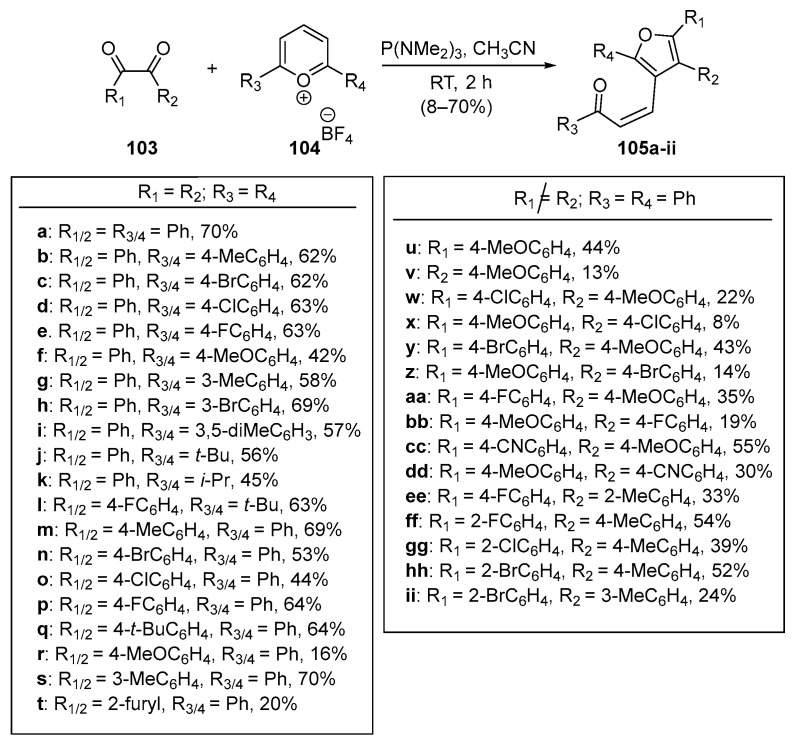
Synthesis of furanyl hybrid Z-chalcones via pyrilium ring-opening benzil-derivative reductive 3+2 annulation.

Tan and Wang leveraged a similar pyrilium ring-opening strategy in a single-pot 3+2 reductive annulation with benzil derivatives **103** to prepare a comprehensive library of tetra-substituted Furano chalcones **105a–ii** in yields as high as 70% [65]. See Scheme 33. A noteworthy observation in both works was the finding that *Z*-chalcone derivatives were the major or sole product in all instances.

## 4. A–B Ring Dual Heteroaromatic Hybrid Chalcone Synthesis

This section catalogues selected processes by which hybrid chalcones bearing a heteroaromatic species at both rings A and B may be prepared. Of particular note is the incredibly diverse array of chalcones produced that feature 21 different heteroaromatic A–B ring-substituted groups on the hybrid chalcones shown in Scheme 34, Scheme 35, Scheme 36, Scheme 37, Scheme 38, Scheme 39, Scheme 40, Scheme 41, Scheme 42, Scheme 43, Scheme 44, Scheme 45 and Scheme 46.

### 4.1. Claisen–Schmidt Condensations

As noted in the preceding sections, the Claisen–Schmidt (C-S) condensation is the most common method used to prepare A–B ring heteroaromatic chalcones. This reaction, which can be catalyzed by bases or acids, offers mild conditions that tolerate a wide scope of functionality in both the ketone donors and aldehyde acceptors.

#### 4.1.1. Base-Catalyzed C-S Condensations 

**Scheme 34:**
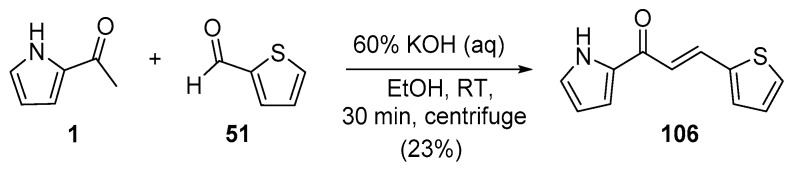
Synthesis of pyrrolyl–thienyl hybrid chalcones.

**Scheme 35:**
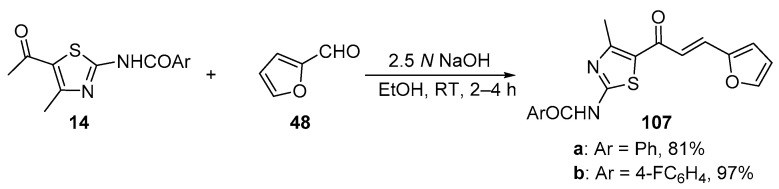
Synthesis of thiazolyl–furyl hybrid chalcones.

In most instances, NaOH and KOH are the most widely used bases. Sweeting’s group synthesized and obtained an X-ray crystal structure for the pyrrolyl–thienyl hybrid chalcone **106** as part of a chalcone solubility and stability study [30]. See Scheme 34. While the use of centrifuging to mix the reagents is of interest, the low yield is likely attributable to the limited reaction time of 30 min. Sinha and coworkers prepared two thiazolyl–furyl hybrid chalcones in high yields (Scheme 35) while investigating potential ant-lipoxygenase agents [37].

**Scheme 36:**
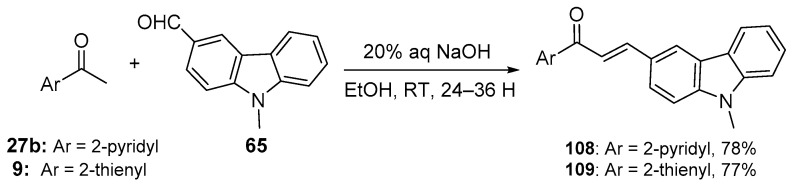
Synthesis of pyridyl– and thienyl–carbazole hybrid chalcones.

**Scheme 37:**
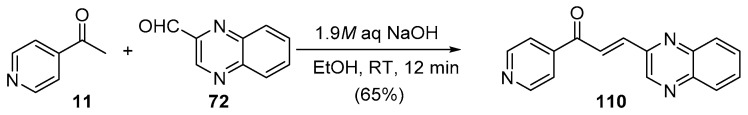
Synthesis of pyridyl–quinoxazolyl hybrid chalcone.

**Scheme 38:**
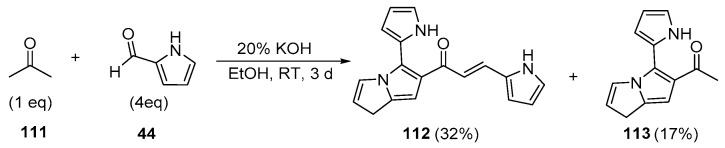
Synthesis of pyrrole–[(2-pyrrolyl)-3H-pyrrolizinyl] hybrid chalcone.

**Scheme 39:**
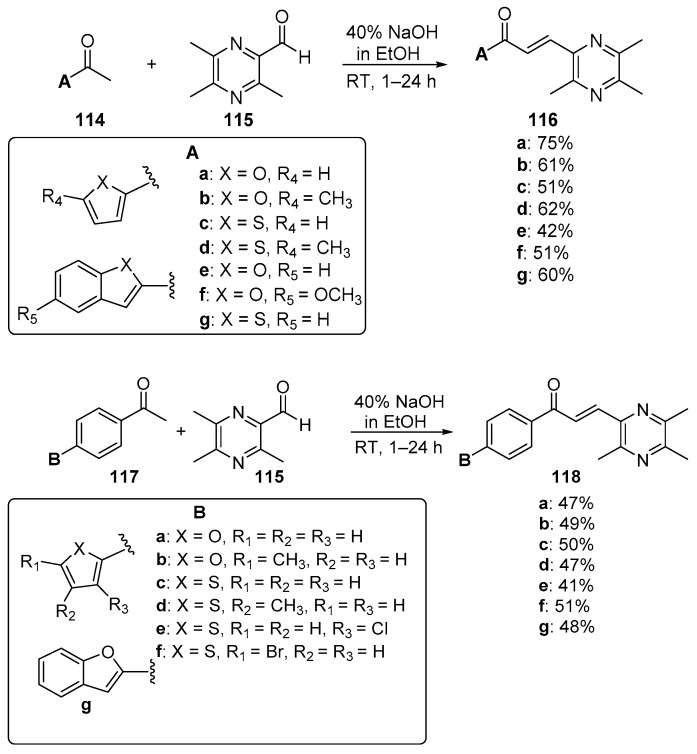
Synthesis of pyrazinyl hybrid chalcones.

Fused-ring A–B hybrid chalcone examples have also been successfully prepared under very mild, base-catalyzed C-S conditions. Bandgar’s team prepared the pyridyl and thienyl–carbazolyl heteroaromatic hybrid chalcones **108–109** in very good yields (Scheme 36) [29]. While investigating ACP reductase inhibition, Desai’s group prepared the pyridyl/quinoxazolyl chalcone **110** in a good yield as shown in Scheme 37 [23]. Mallik et al. found that when one equivalent of acetone and four equivalents of 2-pyrrole carbaldehyde were condensed in 20% KOH, the unusual pyrrolizinyl–pyrrolyl chalcone **112** was formed in modest yield (32%), accompanied by the acetylpyrrolizine **113** (17%) [53]. See Scheme 38. This finding is complementary to the work shown in Scheme 25 in which similar pyrrolizine products were formed. In an examination of chalcones with potential anticancer properties, Bukhari prepared a diverse set of furyl-, thienyl-, benzofuryl, and benzothienyl-1,4-pyrazinyl chalcones **116** in yields ranging from 42 to 75%. Extending that work to include condensations of 4-heteroaromatic acetophenones **117** with pyrazine carbaldehyde **115** gave rise to an array of hybrid chalcones **118** in moderate yields [18]. See Scheme 39.

#### 4.1.2. Green C-S Condensations 

**Scheme 40:**
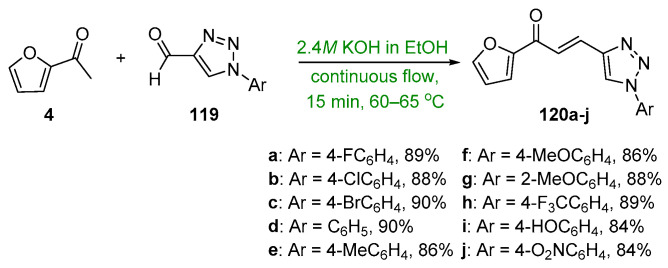
Synthesis of furyl–triazolyl hybrid chalcones.

**Scheme 41:**
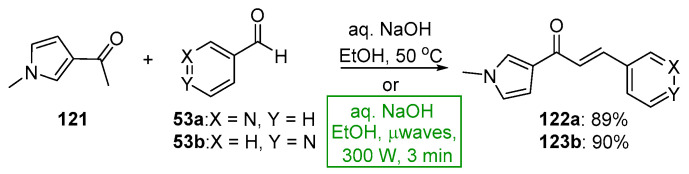
Synthesis of pyrrolyl–pyridyl hybrid chalcones.

The recent literature reports a number of green, base-promoted C-S condensations used to prepare A–B ring heteroaromatic hybrid chalcones. While studying potential antimicrobial agents, Kumar et al. synthesized ten furyl-triazolyl chalcones **120a–j** via a continuous-flow reactor [66]. Of note are the exceptional yields (84–90%) obtained in only 15 min. See Scheme 40. Moreover, in pursuit of suitable chalcones that have antimicrobial properties, Usta’s team prepared two pyrrole–pyridyl chalcones using both conventional and microwave processes [27]. The yields reported were as high as 90% after only 3 min of irradiation. See Scheme 41.

**Scheme 42:**
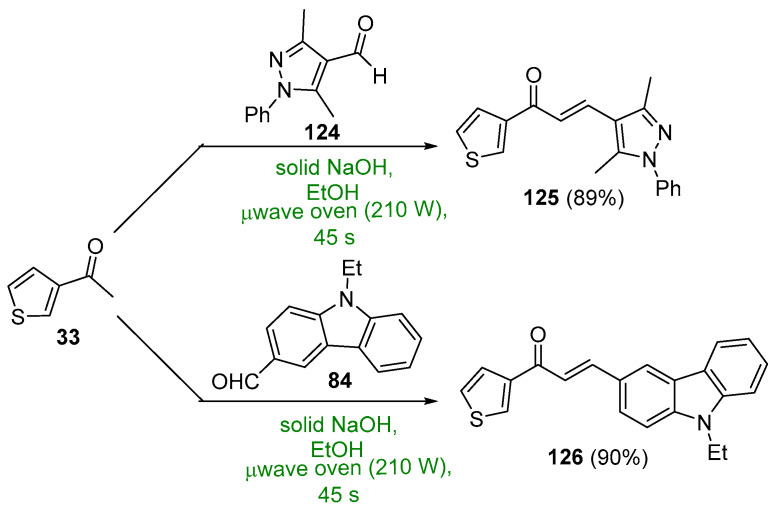
Synthesis of thienyl–pyrazolyl/carbazolyl hybrid chalcones.

**Scheme 43:**
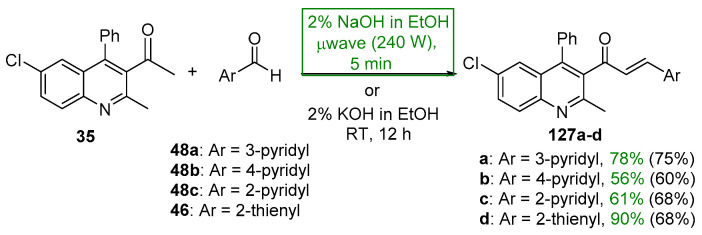
Synthesis of quinolinyl–pyridyl/thienyl hybrid chalcones.

Several syntheses of A–B ring heteroaromatic chalcones having fused-ring systems have also been reported. Khan and Asiri prepared two hybrid chalcones and tested them for antibacterial activity, a thienyl–pyrazole chalcone as well as a thienyl–carbazolyl chalcone using a microwave oven [46]. See Scheme 42. The base-catalyzed process, completed in only 45 s, provided the chalcones in 89–90%. Quinolinyl chalcones, such as those prepared by Sarveswari and Vijayakumar in Scheme 43, have also shown promise as antibacterial and antifungal agents [47]. Again, yields for the short-duration, microwave-mediated process was on par with or exceeded those obtained by the conventional C-S reactions conducted in their comparative study.

**Scheme 44:**
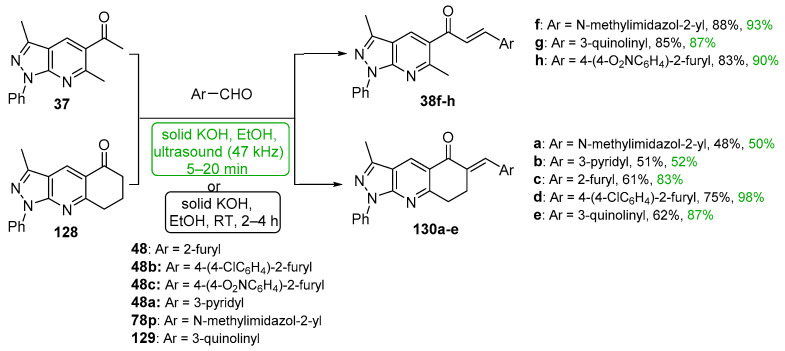
Synthesis of pyrazolopyridyl–heteroaryl hybrid chalcones.

Acetylated pyrazolo pyridines **37** and **128** were condensed with five heteroaryl aldehydes by Polo et al. under both ultrasonic and conventional conditions to prepare interesting A–B ring hybrid chalcones substituted with furyl, pyridyl, imidazolyl and quinolinyl groups [48]. See Scheme 44. Chalcone series **38** was part of a larger study discussed earlier in the review (Scheme 17). Yields for the short-duration ultrasound-assisted condensation met or exceeded those obtained by the conventional, base-promoted C-S condensations performed by the group.

**Scheme 45:**
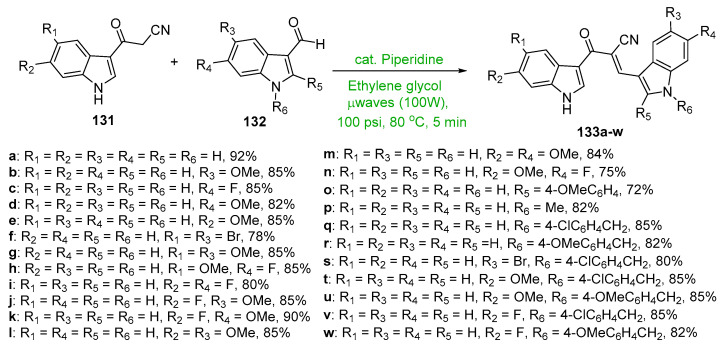
Synthesis of twin indolyl hybrid chalcones.

In Scheme 45, Kumar et al. employed piperidine base to catalyze the microwave-mediated condensation of indoles **131** and **132** en route to a large array of highly differentially functionalized twin indolyl hybrid chalcones **133** [67]. The yields reported were excellent, ranging from 72 to 92%, especially given the reaction time of 5 min.

**Scheme 46:**
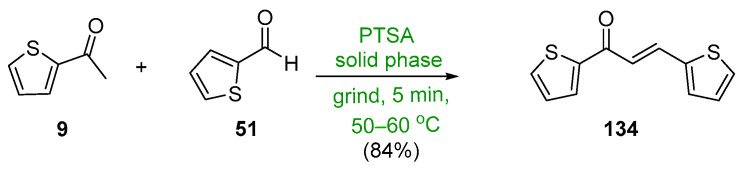
Synthesis of twin thienyl hybrid chalcone.

Our final entry in this section is a green, solid-state, acid-catalyzed condensation of 2-acetylthiophene **9** and the thienyl carboxaldehyde **51** conducted by Adnan and associates, which produced the twin thienyl chalcone **134** in an excellent yield [13]. See Scheme 46.

## 5. Heteroaromatic Bis Chalcone Hybrid Synthesis

This section catalogues several processes by which heteroaromatic bis chalcone hybrids bearing two or more heteroaromatic species have been prepared. The reactions feature both heteroaromatic donors and acceptors as the linker unit in the bis hybrid chalcone systems. Conventional and green condensations as well as a unique Wittig preparation are discussed.

### 5.1. Claisen–Schmidt Condensations 

The Claisen–Schmidt (C-S) condensation is the most widely used method to prepare heteroaromatic bis chalcone hybrids. In this section, we present base-promoted condensations that tolerate a wide scope of functionality in both the bis-ketone donors and bis-aldehyde acceptors.

#### 5.1.1. Base-Catalyzed C-S Condensations 

**Scheme 47:**
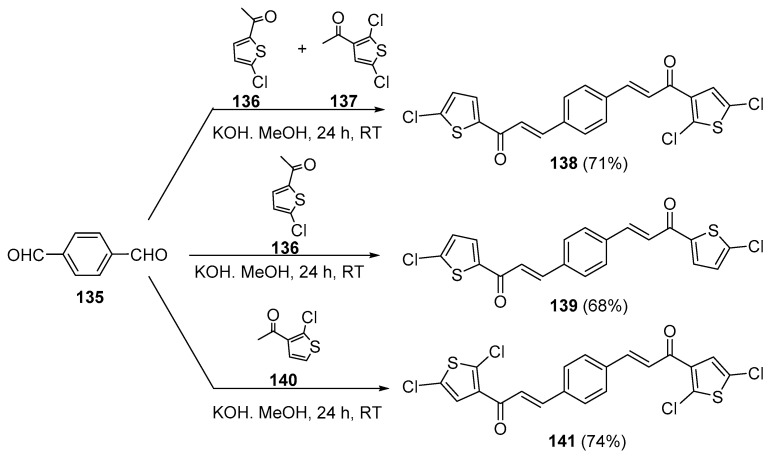
Synthesis of bis thienyl hybrid chalcones.

As seen in the previous sections, NaOH and KOH are the most widely used bases. Methanol and ethanol are the solvents of choice in these condensations. In the first entry of bis hybrid chalcone preparation (Scheme 47), Alidmat et al. prepared three examples of mono- and dichlorinated bis-thienyl chalcones with potential as anticancer agents [68]. Of note is the one-pot preparation of the non-symmetric bis hybrid chalcone **138** from the condensation of 4-formylbenzaldehyde **135** (1 mole) and equimolar quantities of acetylthiophenes **136** and **137**. In contrast, the condensation of **135** (1 mole) with two moles of **136** or **137** resulted in the symmetric bis hybrid chalcones **139** or **141**, respectively.

**Scheme 48:**
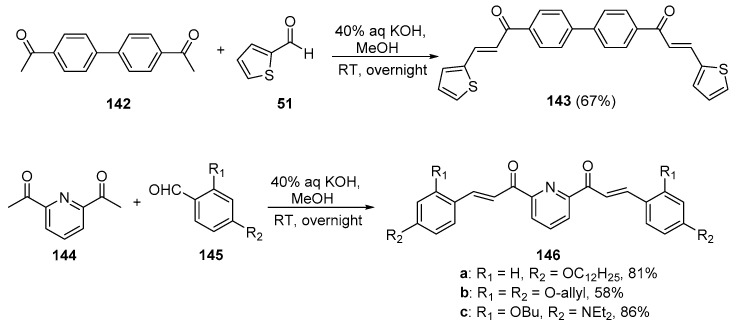
Synthesis of biphenyl bis thienyl and pyridyl bis aryl hybrid chalcones.

While investigating photoinitiators with applications in 3D/4D printing, Chen’s group prepared several bis hybrid chalcones that show promise as light-sensitive photoinitiators. See Scheme 48. 4,4′-diacetylbiphenyl **142** was condensed with 2-formylthiophene under mild, base-promoted conditions to synthesize the bis thienyl biphenyl chalcone **143** in a good yield [17]. Under the same reaction conditions, 2,6-diacetylpyridine **144** was condensed with several substituted benzaldehydes **145** en route to three pyridyl bis aryl hybrid chalcones **146a**–**c** in yields ranging from 58 to 86%.

**Scheme 49:**
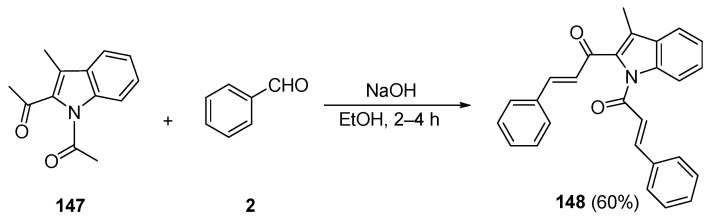
Synthesis of indolyl bis aryl hybrid chalcones.

While investigating lung cancer cell growth inhibitors, Zhao et al. prepared the indole bis phenyl chalcone **148** by condensing 1,2-diacetyl-3-methylindole **147** with benzaldehyde in 60% yield [54]. See Scheme 49.

**Scheme 50:**
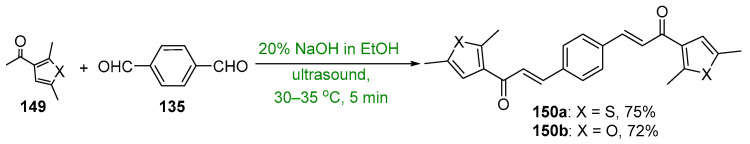
Sonochemical synthesis of bis thienyl and bis furyl hybrid chalcones.

**Scheme 51:**
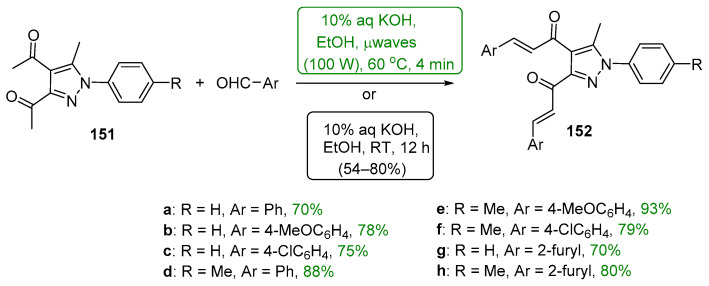
Microwave-mediated synthesis of bis aryl/heteroaryl chalcone pyrazoles.

Presented in Scheme 50 and Scheme 51 are green methods used to prepare bis heteroaromatic chalcones. Asir and coworkers used sonochemical mediation to prepare examples of bis thienyl and bis furyl hybrid chalcones **150a–b**. The reaction time of 5 min was sufficient to give product yields in excess of 70%. [69] In a study of the anti-inflammatory activity of 3,4-bis-chalcone-N-arylpyrazoles, Abdel-Aziz et al. prepared eight examples of assorted aryl- and heteroaryl-substituted chalcone pyrazoles **152** using an aqueous KOH/EtOH medium at 60 °C and microwave irradiation [70]. The total reaction time reported was only four minutes to achieve yields ranging from 70 to 93%. Analogous conventional C-S condensations were also carried out over a 12 h period; the yields obtained were about 75–85% of those obtained with *μ*wave mediation.

#### 5.1.2. Non C-S Condensations

**Scheme 52:**
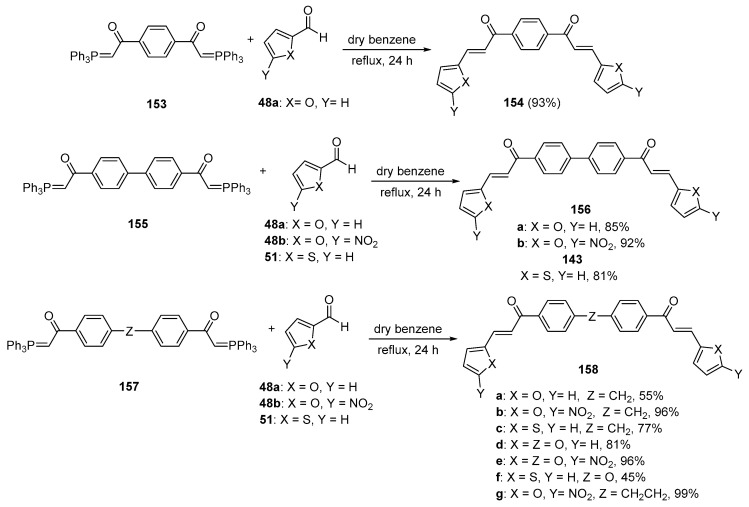
Wittig synthesis of bis thienyl and bis furyl hybrid chalcones.

Our final installment for the bis hybrid chalcone section is an early example published by Saikachi and Muto in 1971 [71]. Their work, shown in Scheme 52, which focused on the preparation and utility of bisphosphoranes in oligimerization studies, exemplified how the bis-Wittig reagents **153**, **155** and **157** could be successfully coupled with furan or thienylcarbaldehydes to provide a series of bis heteroaromatic chalcones **154**, **156** and **158** in yields ranging from 45 to 99%. This work was unique in providing the bis hybrid chalcone system with benzene, biphenyl, diphenyl ether, diphenylmethylene, and diphenylethylene linker units.

## 6. Conclusions and Future Directions

This review of the preparation of heteroaromatic hybrid chalcones gives a robust accounting of more than 50 historic and current synthetic processes leading to more than 430 different hybrid chalcone examples that include single-ring and multi-ring heteroaromatic moieties. We have shown that the venerable Claisen–Schmidt reaction, by far the most common condensation method discussed herein, has been successfully used in either base-promoted or acid-catalyzed processes en route to heteroaromatic hybrid chalcones. We note that variations in the base or acid identity, solution concentration and physical state often make direct comparisons of the yields challenging. Also discussed has been the wide array of reaction conditions, such as the temperature and reaction time, which likewise impact the overall yield. Finally, the topology and electronic reactivity of the ketone donors and aldehyde acceptors likely modulate the product stereochemistry and yields as well.

Additionally, this review has provided the reader with an appreciation of alternative methods used to prepare these hybrid chalcones. Presented in our review are metal-catalyzed coupling reactions, cycloadditions, ring-opening processes and Wittig reactions that enable the formation of more than 75 hybrid chalcone examples.

A key thrust of this review has been to highlight the application of green chemistry methods in heteroaromatic hybrid chalcone synthesis. From the use of benign/renewable solvents and solvent-free and solid-state processes, researchers have demonstrated the ability to minimize waste streams. Through the use of sonochemical, mechanochemical, microwave irradiation, continuous-flow reactions and nanocatalytic methods, scientists minimize the reagent costs, reaction times and energy expenditure while optimizing the yields. Taken together, the important advances in green method uses noted herein portend well for future investigations of heteroaromatic chalcone synthesis.

## Data Availability

Not applicable.

## References

[1] Kostanecki S., Tambor J. (1899). Ueber die sechs isomeren Monooxybenzalacetophenone (Monooxychalkone). J. Chem. Ber..

[2] Faass M., Mallia A., Sommer R., Sloop J. (2021). (Z)-2-(3, 5-Dimethoxybenzylidene) Indan-1-one. CCDC 2081969: Experimental Crystal Structure Determination. CSD Commun..

[3] Encarnacion-Thomas E., Sommer R.D., Mallia V.A., Sloop J. (2020). (E)-2-(3,5-Dimethoxy-benzylidene)indan-1-one. IUCrData.

[4] Makarov A., Sorotskaja L., Uchuskin M., Trushkov I. (2016). Synthesis of quinolines via acid-catalyzed cyclodehydration of 2-(tosylamino)chalcones. Chem. Heterocycl. Comp..

[5] Jin H., Jiang X., Yoo H., Wang T., Sung C., Choi U., Lee C.-R., Yu H., Koo S. (2020). Synthesis of Chalcone-Derived Heteroaromatics with Antibacterial Activities. ChemistrySelect.

[6] Kalirajan R., Sivakumar S., Jubie S., Gowramma B., Suresh B. (2009). Synthesis and Biological evaluation of some heterocyclic derivatives of Chalcones. Int. J. ChemTech Res..

[7] El-Gohary N. (2014). Arylidene Derivatives as Synthons in Heterocyclic Synthesis. Open Access Lib. J..

[8] Albuquerque H., Santos C., Cavaleiro J., Silva A. (2014). Chalcones as Versatile Synthons for the synthesis of 5- and 6-membered Nitrogen Heterocycles. Curr. Org. Chem..

[9] Zhuang C., Zhang W., Sheng C., Zhang W., Xing C., Miao Z. (2017). Chalcone: A Privileged Structure in Medicinal Chemistry. Chem. Rev..

[10] Ardiansah B. (2019). Chalcones bearing N, O, and S-heterocycles: Recent notes on their biological significances. J. Appl. Pharm. Sci..

[11] Sharshira E., Hamada N. (2012). Synthesis and Antimicrobial Evaluation of Some Pyrazole Derivatives. Molecules.

[12] Borge V.V., Patil R.M. (2023). Comparative Study on Synthesis and Biological, Pharmaceutical Applications of Aromatic Substituted Chalcones. Mini. Rev. Org. Chem..

[13] Mhaibes R.M. (2023). Antimicrobial and Antioxidant Activity of Heterocyclic Compounds Derived from New Chalcones. J. Med. Chem. Sci..

[14] Adnan D., Singh B., Mehta S., Kumar V., Kataria R. (2020). Simple and solvent free practical procedure for chalcones: An expeditious, mild and greener approach. Curr. Res. Green Sustain. Chem..

[15] Urbonavîcius A., Fortunato G., Ambrazaitytė E., Plytninkienė E., Bieliauskas A., Milišíunaitė V., Luisi R., Arbâciauskienė E., Krikštolaitytė S., Šâckus A. (2022). Synthesis and Characterization of Novel Heterocyclic Chalcones from 1-Phenyl-1H-pyrazol-3-ol. Molecules.

[16] Kitawata B., Singha M., Kale R. (2013). Solvent Free Synthesis, Characterization, Anticancer, Antibacterial, Antifungal, Antioxidant and SAR Studies of Novel (E)-3-aryl-1-(3-alkyl-2-pyrazinyl)-2-propenone. New J. Chem..

[17] Chen H., Noirbent G., Liu S., Brunel D., Graff B., Gigmes D., Zhang Y., Sun K., Morlet-Savary F., Xiao P. (2021). Bis-Chalcone Derivatives Derived from Natural Products as Near-UV/Visible Light Sensitive Photoinitiators for 3D/4D Printing. Mater. Chem. Front..

[18] Mara Silva de Padua G., Maria De Souza J., Celia Moura Sales M., Gomes de Vasconcelos L., Luiz Dall’Oglio E., Faraggi T.M., Moreira Sampaio O., Campos Curcino Vieira L. (2021). Evaluation of Chalcone Derivatives as Photosynthesis and Plant Growth Inhibitors. Chem. Biodivers..

[19] Bukhari S. (2022). Synthesis and evaluation of new chalcones and oximes as anticancer agents. RSC Adv..

[20] Gaber M., El-Ghamry H.A., Mansour M.A. (2018). Pd(II) and Pt(II) chalcone complexes. Synthesis, spectral characterization, molecular modeling, biomolecular docking, antimicrobial and antitumor activities. J. Photochem. Photobiol. A Chem..

[21] Baretto M., Fuchi N. (2013). Tissue Transglutaminase Inhibitor, Chalcone Derivative, and Pharmaceutical Application Thereof. Japan Patent.

[22] Özdemir A., Altıntop M.D., Turan-Zitouni G., Çiftçi G.A., Ertorun I., Alataş Ö., Kaplancıklı Z.A. (2015). Synthesis and evaluation of new indole-based chalcones as potential antiinflammatory agents. Eur. J. Med. Chem..

[23] Shaik A., Bhandare R., Palleapati K., Nissankararao S., Kancharlapalli V., Shaik S. (2020). Antimicrobial, Antioxidant, and Anticancer Activities of Some Novel Isoxazole Ring Containing Chalcone and Dihydropyrazole Derivatives. Molecules.

[24] Desai V., Desai S., Gaonkar S.N., Palyekar U., Joshi S.D., Dixit S.K. (2017). Novel quinoxalinyl chalcone hybrid scaffolds as enoyl ACP reductase inhibitors: Synthesis, molecular docking and biological evaluation. Bioorg. Med. Chem. Lett..

[25] Gomes M.N., Braga R.C., Grzelak E.M., Neves B.J., Muratov E.N., Ma R., Klein L.K., Cho S., Oliveira G.R., Franzblau S.G. (2017). QSAR-driven design, synthesis and discovery of potent and selective chalcone derivatives with antitubercular activity. Eur. J. Med. Chem..

[26] Hawash M., Kahraman D.C., Eren F., Atalay R.C., Baytas S.N. (2017). Synthesis and biological evaluation of novel pyrazolic chalcone derivatives as novel hepatocellular carcinoma therapeutics. Eur. J. Med. Chem..

[27] Minders C., Petzer J., Petzer A., Lourens A. (2015). Monoamine oxidase inhibitory activities of heterocyclic chalcones. Bioorg. Med. Chem. Lett..

[28] Usta A., Oztürk E., Beriş F. (2014). Microwave-assisted preparation of azachalcones and their N-alkyl derivatives with antimicrobial activities. Nat. Prod. Res..

[29] Li P., Jiang H., Zhang W., Li Y., Zhao M., Zhou W. (2018). Synthesis of carbazole derivatives containing chalcone analogs as non-intercalative topoisomerase II catalytic inhibitors and apoptosis inducers. Eur. J. Med. Chem..

[30] Bandgar B., Adsul L., Lonikar S., Chavan H., Shringare S., Patil S., Jalde S., Koti B., Dhole N., Gacche R. (2013). Synthesis of novel carbazole chalcones as radical scavenger, antimicrobial and cancer chemopreventive agents. J. Enzym. Inhib. Med. Chem..

[31] Sweeting S., Hall C., Potticary J., Pridmore N., Warren S., Cremeens M., D’Ambruoso G., Matsumoto M., Hall S. (2020). The solubility and stability of heterocyclic chalcones compared with transchalcone. Acta Cryst. B.

[32] Robinson T.P., Hubbard R.B., Ehlers T.J., Arbiser J.L., Goldsmith D.J., Bowen J.P. (2005). Synthesis and biological evaluation of aromatic enones related to curcumin. Bioorg. Med. Chem..

[33] Song T., Duan Y., Yang Y. (2019). Chemoselective transfer hydrogenation of α,β-unsaturated carbonyls catalyzed by a reusable supported Pd nanoparticles on biomass-derived carbon. Catal. Commun..

[34] Lokeshwari D.M., Rekha N.D., Srinivasan B., Vivek H.K., Kariyappa A.K. (2017). Design, synthesis of novel furan appended benzothiazepine derivatives and in vitro biological evaluation as potent VRV-PL-8a and H+/K+ ATPase Inhibitors. Bioorg. Med. Chem. Lett..

[35] Liu W., Shi H.-M., Jin H., Zhao H.-Y., Zhou G.-P., Wen F., Yu Z.-Y., Hou T.-P. (2009). Design, Synthesis and Antifungal Activity of a Series of Novel Analogs Based on Diphenyl Ketones. Chem. Biol. Drug. Des..

[36] Robinson S.J., Petzer J.P., Petzer A., Bergh J.J., Lourens A.C.U. (2013). Selected furanochalcones as inhibitors of monoamine oxidase. Bioorg. Med. Chem. Lett..

[37] Parveen H., Iqbal P.F., Azam A. (2008). Synthesis and Characterization of a New Series of Hydroxy Pyrazolines. Synth. Commun..

[38] Sunduru N., Agarwal A., Katiyar S.B., Goyal N., Gupta S., Chauhana P.M.S. (2006). Synthesis of 2,4,6-trisubstituted pyrimidine and triazine heterocycles as antileishmanial agents. Bioorg. Med. Chem..

[39] Sinha S., Manju S., Doble M. (2019). Chalcone-Thiazole Hybrids: Rational Design, Synthesis, and Lead Identification against 5-Lipoxygenase. Med. Chem. Lett..

[40] Zhao X., Dong W., Gao Y., Shin D.-S., Ye Q., Su L., Jiang F., Zhao B., Miao J. (2017). Novel indolyl-chalcone derivatives inhibit A549 lung cancer cell growth through activating Nrf-2/HO-1 and inducing apoptosis in vitro and in vivo. Sci. Rep..

[41] Hsieh C.-Y., Ko P.-W., Chang Y.-J., Kapoor M., Liang Y.-C., Chu H.-L., Lin H.-H., Horng J.-C., Hsu M.-H. (2019). Design and Synthesis of Benzimidazole-Chalcone Derivatives as Potential Anticancer Agents. Molecules.

[42] Hsieh C., Kuiying Xu K., Lee I., Graham T., Tu Z., Dhavale D., Kotzbauer P., Mach R. (2018). Chalcones and Five-Membered Heterocyclic Isosteres Bind to Alpha Synuclein Fibrils in Vitro. ACS Omega.

[43] Saito Y., Kishimoto M., Yoshikawa Y., Kawaii S. (2015). Synthesis and Structure–Activity Relationship Studies of Furan-ring Fused Chalcones as Antiproliferative Agents. Anticancer Res..

[44] Grigoropoulou S., Manou D., Antoniou A.I., Tsirogianni A., Siciliano C., Theocharis A.D., Athanassopoulos C.M. (2022). Synthesis and Antiproliferative Activity of Novel Dehydroabietic Acid-Chalcone Hybrids. Molecules.

[45] Mubofu E.B., Engberts J.B.F.N. (2004). Specific acid catalysis and Lewis acid catalysis of Diels–Alder reactions in aqueous media. J. Phys. Org. Chem..

[46] Jianga X., Jina H., Wanga T., Yoob H., Koo S. (2019). Synthesis of Phenyl-2,2-bichalcophenes and Their Aza-Analogues by Catalytic Oxidative Deacetylation. Synthesis.

[47] Ritter M., Martins R., Rosa S., Malavolta J., Lund R., Flores A., Pereira C. (2015). Green Synthesis of Chalcones and Microbiological Evaluation. J. Braz. Chem. Soc..

[48] Khan S., Asiri A. (2013). Green Synthesis, Characterization and biological evaluation of novel chalcones.as anti bacterial agents. Arab. J. Chem..

[49] Sarveswari S., Vijayakumar V. (2016). A rapid microwave assisted synthesis of 1-(6-chloro-2-methyl-4-phenylquinolin-3-yl)-3-(aryl)prop-2-en-1-ones and their anti bacterial and anti fungal evaluation. Arab. J. Chem..

[50] Polo E., Ferrer-Pertuz K., Trilleras J., Quiroga J., Guti’errez M. (2017). Microwave-assisted one-pot synthesis in water of carbonylpyrazolo [3,4-b]pyridine derivatives catalyzed by InCl3 and sonochemical assisted condensation with aldehydes to obtain new chalcone derivatives containing the pyrazolopyridinic moiety. RSC Adv..

[51] Yadav P., Yadav M., Gaur R., Gupta R., Arora G., Rana P., Srivastava A., Sharma R.K. (2020). Fabrication of copper-based silica-coated magnetic nanocatalyst for efficient one-pot synthesis of chalcones via A3 coupling of aldehydes-alkynes-amines. Chem. Cat. Chem..

[52] Fu D.-J., Zhang S.-Y., Liu Y.-C., Yue X.-X., Liu J.-J., Song J., Zhao R.-H., Li F., Sun H.-H., Zhang Y.-B. (2016). Design, synthesis and antiproliferative activity studies of 1,2,3-triazole–chalcones. Med. Chem. Commun..

[53] Gadhave A.G., Uphade B.K. (2017). Synthesis of Some Pyrazole Containing Chalcones and Pyridine-3-Carbonitriles and Study of their Anti-inflammatory Activity. Orient. J. Chem..

[54] Mallik A., Dey S., Chattopadhyay F., Patra A. (2000). Novel formation of 6-acyl-5-(2-pyrrolyl)-3H-pyrrolizines bym base-catalysed condensation of pyrrole-2-aldehyde with methyl ketones. Tetrahedron Lett..

[55] Bindu P., Mahadevan K., Naik T., Harish B. (2014). Synthesis, DNA binding, docking and photocleavage studies of quinolinyl chalcones. Med. Chem. Commun..

[56] Abonia R., Gutiérrez L., Quiroga J., Insuasty B. (2018). (E)-3-[3-(2-Butoxyquinolin-3-yl)acryloyl]-2-hydroxy-4H-chromen-4-one. Molbank.

[57] Sun M., Yuyang W., Minghua Y., Qing Z., Yixin Z., Yongfang Y., Yongtao D. (2021). Angiogenesis, Anti-Tumor, and Anti-Metastatic Activity of Novel α-Substituted Hetero-Aromatic Chalcone Hybrids as Inhibitors of Microtubule Polymerization. Front. Chem..

[58] Ashok D., Ravi S., Ganesh A., Lakshmi B., Adam S., Murthy S. (2016). Microwave-assisted synthesis and biological evaluation of carbazole-based chalcones, aurones and flavones. Med. Chem. Res..

[59] Bhatt K., Vishal Rana V., Patel N., Parikh J., Pillai S. (2023). Novel Oxygen Fused Bicyclic Derivatives and Antioxidant Labelling: Bioactive Chalcone Based Green Synthesis. Biointerface Res. Appl. Chem..

[60] Jadhava S., Peerzadeb N., Gawalia R., Bhosaleb R., Kulkarnic A., Varpe B. (2020). Green synthesis and biological screening of some fluorinated pyrazole chalcones in search of potent anti-inflammatory and analgesic agents. Egypt Pharmaceut. J..

[61] Kudlickova Z., Stahorsky M., Michalkova R., Vilkova M., Balaz M. (2022). Mechanochemical synthesis of indolyl chalcones with antiproliferative activity. Green Chem. Lett. Rev..

[62] Nimmala S., Chepyala K., Balram B., Ram B. (2012). Synthesis and antibacterial activity of novel imidazo [1,2-a]pyrimidine and imidazo [1,2-a]pyridine chalcone derivatives. Der Pharma Chem..

[63] Joshi H., Saglani M. (2020). Rapid and greener ultrasound assisted synthesis of series (2-((substituted 2-Chloroquinolin-3-yl) methylene)-3,4-dihydronaphthalen-1(2h)-one) derivatives and their biological activity. J. Adv. Sci. Res..

[64] Devi L., Sharma G., Kant R., Shukla S., Rastogi N. (2021). Regioselective Synthesis of Functionalized Pyrazole-Chalcones via Base Mediated Reaction of Diazo Compounds with Pyrylium Salts. Org. Biomol. Chem..

[65] Tan P., Wang S.R. (2019). Reductive (3 + 2) Annulation of Benzils with Pyrylium Salts: Stereoselective Access to Furyl Analogues of Cis-Chalcones. Org. Lett..

[66] Kumar K., Siddaiah V., Lilakar J., Ganesh A. (2020). An efficient continuous-flow synthesis and evaluation of antimicrobial activity of novel 1,2,3-Triazole-Furan hybrid chalcone derivatives. Chem. Data Collect..

[67] Kuamr D., Kumar N.M., Tantak M.P., Ogura M., Eriko Kusaka E., Ito T. (2014). Synthesis and identification of α-cyano bis(indolyl)chalcones as novel anticancer agents. Bioorg. Med. Chem. Lett..

[68] Alidmat M., Khairuddean M., Salhimi S., Al-Amin M. (2021). Docking studies, synthesis, characterization, and cytotoxicity activity of new bis-chalcone derivatives. Biomed. Res. Ther..

[69] Asiri A., Marwani H., Alamry K., Al-Amoudi M., Khan S., El-Daly S. (2014). Green Synthesis, Characterization, Photophysical and Electrochemical Properties of Bis-chalcones. Int. J. Electrochem. Sci..

[70] Abdel-Aziz H.A., Al-Rashood K.A., ElTahir K.E.H., Ibrahim H.S. (2011). Microwave-assisted Synthesis of Novel 3,4-Bis-chalcone-N-arylpyrazoles and Their Anti-inflammatory Activity. J. Chin. Chem. Soc..

[71] Saikachi H., Muto H. (1971). Preparation of Resonance-Stabilized Bisphophoranes. Chem. Pharm. Bull..

